# Two-Step Air/Water Oxidation Process for the Long-Lasting Photoluminescence and Biological Viability (MTT Assay) of Porous Silicon Particles

**DOI:** 10.3390/jox15050168

**Published:** 2025-10-17

**Authors:** Claudia Castillo Calvente, María F. Gilsanz-Muñoz, Javier Pérez-Piñeiro, Arisbel Cerpa-Naranjo, Rodrigo Blasco, Elvira Bragado-García, María S. Fernández-Alfonso, Darío Gallach-Pérez

**Affiliations:** 1 School of Architecture, Engineering and Desing, European University of Madrid, C/Tajo s/n, Villaviciosa de Odón, 28670 Madrid, Spain; claudia.castillo.calvente@gmail.com (C.C.C.); javier.perez4@universidadeuropea.es (J.P.-P.); arisbel.cerpa@universidadeuropea.es (A.C.-N.); rodrigo.blasco@universidadeuropea.es (R.B.); 2 Pluridisciplinary Institute and School of Pharmacy, Universidad Complutense de Madrid, Campus Moncloa, 28040 Madrid, Spain; ebragado@ucm.es (E.B.-G.); marisolf@ucm.es (M.S.F.-A.); 3 Department of Applied Physics, Universidad Autónoma de Madrid, Campus de Cantoblanco, 28049 Madrid, Spain; dario.gallach@uam.es

**Keywords:** porous silicon particles, photoluminescence, chemical stabilization, oxidation, cell viability

## Abstract

Due to their visible photoluminescence (PL) at room temperature, porous silicon particles (PSps) have gained interest for their potential biomedical applications, making them promising biological markers for in vivo or in vitro use. This study explores the PL evolution and stabilization of PSps following a two-step oxidation process involving air annealing and chemical oxidation in deionized water. PS layers were fabricated by electrochemical etching of p^+^-Si wafers and then annealed in air at 300 °C and 600 °C for five minutes. The layers were then stored in deionized water and sonicated to produce PSps. Scanning electron microscopy (SEM) and energy-dispersive X-ray spectroscopy (EDX) were used to analyze the morphology and composition of the particles, and spectrofluorimetry was used to monitor the PL over several weeks. Samples annealed at 300 °C exhibited a transition from nearly complete PL quenching to strong yellow–red emission. In contrast, the 600 °C sample showed no PL emission. The cytotoxicity of the PSps was evaluated using an MTT assay on human endothelial cells (EA.Hy926) with PSps and polyethylene glycol (PEG)-coated PSps at concentrations of (3.5–125 µg/mL) in both serum-free and fetal bovine serum (FBS)-containing media over 24, 48, and 72 h. Cell viability was significantly affected by both exposure time and particle concentration; however, this effect was prevented under conditions mimicking the physiological plasma environment.

## 1. Introduction

Porous silicon (PS) is a nanostructured network of silicon (Si) nanocrystals with a high specific surface area [1]. This structure gives PS interesting chemical and physical properties, making it an attractive material for a wide range of applications. Its biocompatibility [2], biodegradability [3] and strong yellow–red photoluminescence (PL) at room temperature [4] make PS particularly interesting for biotechnology applications. Due to these properties, research has focused on the potential of PS as a drug delivery system and as an in vivo or in vitro biological marker [3,5]. For these applications, it is important to maintain PL for as long as possible and understand how it evolves within the medium in which the particles will be used.

The origin of PS PL has been a topic of controversy for a long time, but recent consensus attributes its PL emission to quantum confinement effects [6,7], although models based on surface-bound species, such as oxyhydride-like emitters, silanones, and silylenes, have also provided valuable insights into the PL mechanisms [8,9]. According to the quantum model, Si quantum dots (QDs) are formed within a PS layer during the electrochemical etching of monocrystalline wafers in HF solutions. The surface of the PS layer is covered with highly reactive Si-H bonds [10], leading to rapid surface oxidation in oxidizing media and the formation of silicon suboxides (SiO_x_, where 0 ≤ x ≤ 2). This oxidation process creates a protective oxide layer on the surface that shields the crystalline silicon quantum dots (Si QDs) from further oxidation, depending on the quality of the oxide. However, some Si atoms from the QD crystalline structure will bond with the oxide layer, reducing the size of the QDs and causing changes in the PL or its partial or complete quenching [6,11].

Different methods have been studied to maintain and stabilize the PS PL, including thermal, electrochemical, and chemical techniques [12,13]. Among these methods, thermal annealing of PS layers in air [11,14] appears to be the most promising approach to extending the lifespan of the red–yellow PL and stabilizing it over longer periods. This technique effectively passivates the Si-H bonds covering the PS surface.

In the field of biotechnology, PS is preferred in the form of particles (PSps), which are created by sonicating the PS layer. Therefore, the exposure of this porous material to oxidizing agents is increased, and the effects of annealing on the PS layers and subsequent storage conditions must be examined in greater detail. Our initial hypothesis suggests that that the annealing process of PS layers in air can lead to uneven oxygen distribution. This is due to O_2_ particles diffusing in the porous matrix, resulting in a layer with an oxidation gradient. Thus, when PSps are formed after sonication of the PS layer, they exhibit different degrees of oxidation, which affects their interaction with oxidizing media. Therefore, the particles require another oxidation process to complete a protective silicon oxide layer, which preserves the PSps PL. To test this hypothesis, PS layers were prepared under etching conditions that showed strong PL. Then, the formation of PSps underwent a two-step oxidation process: thermal annealing in air at two different temperatures (300 °C and 600 °C) and sonication in deionized water, known to produce further oxidation of the PS layers [10]. The morphology and initial oxidation of the PS layers after annealing were studied using electron microscopy and energy-dispersive X-ray spectroscopy. Spectrofluorimetry was used to track the PL evolution over several weeks.

The development of silicon-based particles as promising drug delivery carriers and/or in vivo biological markers requires determining their effects on cell viability as a first safety step. The endothelial cell layer in blood vessels is one of the first biological barriers that PSps will encounter after entering the bloodstream via intravenous injection or after systemic distribution from other administration routes. Damage or disruption of this layer leads to endothelial dysfunction impairing vasodilation or clotting balance, as well as to inflammation and oxidative stress promoting vascular injury and atherosclerosis [15]. Since cytotoxicity assays in cell culture give a rapid, controlled, and relatively inexpensive way to identify potential harmful effects of these particles before moving to animal or human studies, we used a human endothelial cell line (EaHy926), previously used in other cytotoxicity studies of silicon particles [15,16,17,18]. Thus, dose- and time-dependent cytotoxicity, as well as the effect of PEGylation, are evaluated in cell culture.

## 2. Materials and Methods

### 2.1. Synthesis and Characterization of Porous Silicon Particles (PSps)

The PS layers were produced by the electrochemical etching of p^+^-type (B-doped) Si wafers with low-resistivity (0.01 < ρ < 0.02 Ω·cm) and (100) orientation. The etching was conducted in a hydrofluoric acid (HF St. Louis, MO, USA, Sigma Aldrich) (48%wt): ethanol absolute > 99.8% (EtOH, St. Louis, MO, USA, Sigma Aldrich) solution (1:2) at room temperature, without any additional illumination. A constant current density of 120 ± 9 mA·cm^−2^ was applied during 30 ± 2 min, resulting in 22.4 ± 0.3 μm-thick PS layers.

After the electrochemical etching, the first oxidation step occurs by annealing the samples in air in a muffle furnace. Based on the existing literature, two different annealing temperatures (300 ± 15 °C and 600 ± 15 °C) were tested to compare the results, since it has been shown that isochronal annealing at temperatures below 300 °C typically induces minimal changes in photoluminescence (PL) [19], and vacuum annealing at temperatures below 600 °C degrades PL intensity [20]. In both cases, the annealing time was 5.0 ± 0.5 min. This process aims to create silicon suboxides (SiO_x_, 0 ≤ x ≤ 2) to produce an initial protective oxide layer.

The morphology of the PS layers was examined by scanning electron microscopy (SEM) using a Hitachi S-3000N (Tokyo, Japan) and a Raith eLine Plus electron microscope, operating at 20 kV and 15 kV, respectively. The chemical composition of the PS layers was analyzed using energy dispersive X-ray spectroscopy (EDX) using elemental mappings from a Quantax EDS model XFlash 7 by Bruker (Karlsruhe, Germany). These images were obtained without prior metallization.

**Figure 1:**
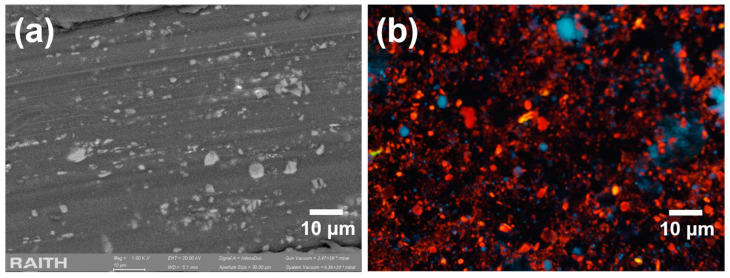
(**a**) SEM image of an aliquot of the PSps dispersion after sonication of the PS layers. (**b**) Same aliquot under UV light.

The PS layers were sonicated in vials filled with deionized water, resulting in porous silicon particles (PSPs) with an average size of 1080 nm and a standard deviation of 670 nm, as shown in Figure 1a. The average size and standard deviation were obtained by analyzing 213 particles shown in Figure 1a using ImageJ software v1.53 (Bethesda, MD, USA). The resulting dispersion was then centrifuged, and the liquid phase was removed and replaced with new deionized water to eliminate any residual HF. These particles were luminescent at room temperature under ultraviolet (UV) illumination (Figure 1b).

PL from the PSps was measured over time using a spectrofluorometer (FP-8300 from Jasco, Yorkshire, UK) with an excitation wavelength of 365.0 ± 0.1 nm from a Xe lamp. The spectra were measured between 500 and 750 nm with data intervals of 0.2 nm, and the scan velocity was set at 100 nm/min. Both excitation and emission bandwidth parameters were set at 5 nm. No additional accessories were placed in the optical path between the sample and the lamp or the photomultiplier tube.

### 2.2. Cell Viability Assay in Presence of Porous Silicon Particles (PSps)

The cytotoxicity of PSps was evaluated using the MTT assay [21] on the EA.hy926 human endothelial cell line (information is archived in the Cellosaurus database under accession number CVCL_3901, catalog number CRL-2922, American Type Culture Collection (ATCC), Manassas, VA, USA), which is a standardized in vitro model of human vascular endothelium widely used for nanoparticle biocompatibility testing [15,16,17,18]. The MTT assay is a colorimetric method that measures cell metabolic activity as an indicator of viability. Viable cells reduce the yellow tetrazolium salt MTT (3-(4,5-dimethylthiazol-2-yl)-2,5-diphenyltetrazolium bromide) into insoluble purple formazan crystals via mitochondrial enzymes. After solubilization of the crystals, the absorbance at 570 nm is directly proportional to the number of viable cells.

Cells were seeded at a density of 1 × 10^4^ cells per well in 96-well plates and cultured overnight in Dulbecco’s Modified Eagle Medium (DMEM; Gibco, Thermo Fisher Scientific Iberia, Madrid, Spain), supplemented with 100 U/mL penicillin and 100 μg/mL streptomycin (Sigma-Aldrich, Merck KGaA, Darmstadt, Germany) at 37 °C in a humidified atmosphere with 5% CO_2_, either in the absence or presence of 10% fetal bovine serum (FBS; Thermo Fisher Scientific Iberia, Madrid, Spain). The following day, cells were exposed to aliquots of PSps or PEGylated PSps (PSps-PEG) at concentrations ranging from 3.5 to 125 μg/mL for 24, 48, or 72 h. Untreated wells served as negative controls (100% viability), while wells exposed to dimethyl sulfoxide (DMSO, Panreac, Madrid, Spain) were used as positive controls (0% cell viability). After exposure to PSps, the medium containing particles was fully aspirated, and the cells were gently washed two to three times with pre-warmed phosphate-buffered saline (PBS) to remove the residual particles. Fresh particle-free DMEM was then added prior to MTT incubation. The cells were then incubated with an MTT solution (5 mg/mL; Sigma-Aldrich, Merck KGaA, Darmstadt, Germany) for 4 h at 37 °C. The formazan crystals were subsequently dissolved in DMSO, and the absorbance was measured at 570 nm using a microplate reader (ELx808, Bio-tek, Shoreline, WA, USA).

Cell viability (%) was calculated relative to the untreated controls, after correcting for optical interference. To this end, particle-only blanks (wells containing particles and MTT but no cells) were included, and their absorbance values were subtracted from the corresponding sample wells. Because the particles were prepared directly in DMEM, the same culture medium as the controls, no additional vehicle control was required. All measurements were performed in duplicate from three independent experiments. For statistical analysis, continuous variables were compared by one-way ANOVA with the Newman–Keuls test. Data analysis was performed with GraphPad Prism 9 software (San Diego, CA, USA). Data are presented as the mean ± standard error of the mean (SEM), and statistical significance was considered for *p* < 0.05.

## 3. Results and Discussion

### 3.1. Photoluminescence Evolution of Porous Silicon Particles (PSps)

**Figure 2:**
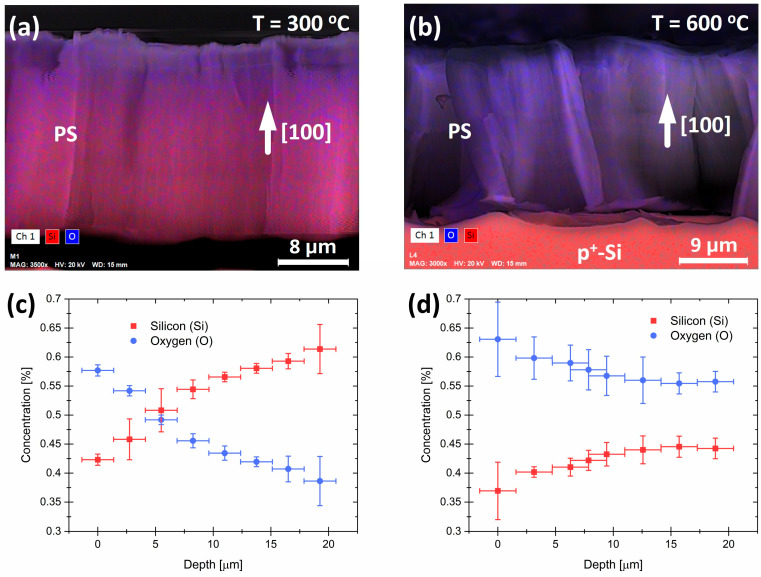
(**a**,**b**) are scanning electron microscopy images with EDX mapping performed. The percentual concentration of Si and O atoms as a function of depth is displayed in the RGB analysis of the EDX mappings, as shown in (**c**,**d**).

Figure 2a,b display cross-sectional SEM images of the PS layers annealed in air at 300 °C and 600 °C, respectively. The colors in the images represent the different atoms present in the layers. Blue represents oxygen (O) atoms, and red represents silicon (Si) atoms, as determined by EDX analysis. In contrast to the sample annealed at 600 °C, the sample annealed at 300 °C shows a higher concentration of O atoms at the surface, indicating that the PS layer was not evenly oxidized. As a comment to both pictures, the p^+^ substrate appears in Figure 2b and not in Figure 2a, since the latter was detached from the substrate.

**Figure 3:**
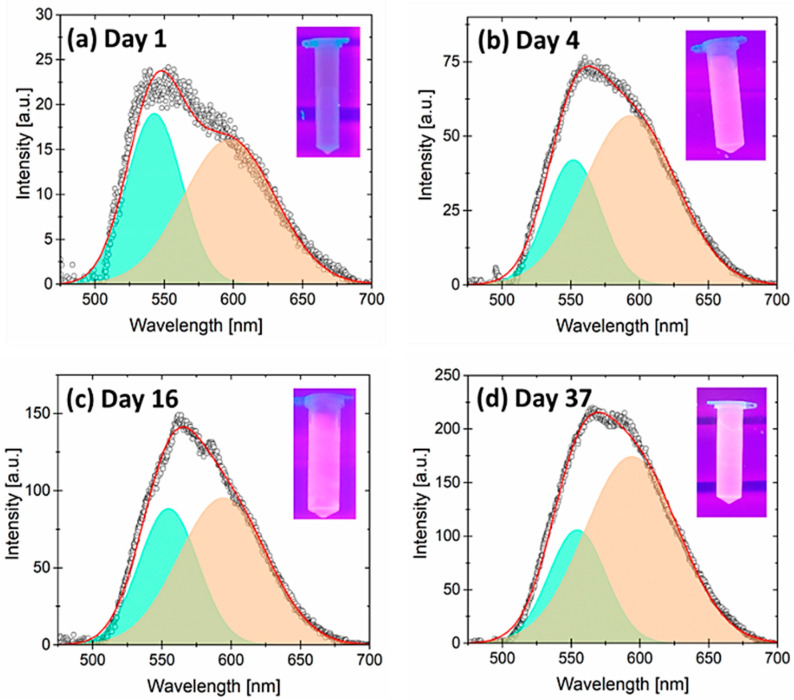
(**a**–**d**) PL emission spectra from the PSps measured on different days (background subtracted). The PL spectra are deconvoluted into two contributions: one yellow–green emission and one yellow–red emission. The insets on these graphs correspond to the emission of the PSps as seen with the naked eye.

Figure 2c,d display the concentrations of O and Si atoms in relation to the depth of the PS layer. The samples were annealed at 300 °C and 600 °C, respectively, and the measured concentrations were determined through an RGB analysis of Figure 2a,b. For instance, in the case of the sample annealed at 300 °C, the ratio between the Si and O percent concentrations [%O]/[%Si] at the surface is approximately 1.35, whereas at the bottom of the layer, this ratio is closer to 0.61. However, if we consider a hypothetical layer entirely formed by SiO_2_, we expect a ratio of about 2. This suggests the presence of Si atoms that are not bonded to O in these samples, which indicates the formation of Si nanocrystals. The concentration of Si atoms is consistently higher than the concentration of oxygen atoms at depths below 5 μm, suggesting a larger number of Si atoms that are not oxidized. In contrast, the ratio in the sample annealed at 600 °C is around 1.70 at the surface and 1.28 at the bottom of the layer. This indicates that the chemistry of the PS layer surface is like that of a stoichiometric silicon oxide layer. Additionally, the oxygen concentration is consistently higher than the silicon concentration, with virtually no variation below 12 μm. These results confirm the effects of oxygen diffusion inside the polystyrene matrix during the annealing process and support the initial hypothesis of inhomogeneous oxygen diffusion along the pores. To produce the PSps, PS layers were stored in deionized water (as shown in Figure 3) and sonicated in an ultrasonic bath. Therefore, the initial degree of oxidation of the PSps is expected to vary based on their original position in the PS layer during the annealing process in air. Since the PSps are dissolved in water, further chemical oxidation is expected to form a protective oxide, which can be observed through the PSps PL spectra.

Figure 3 shows the PL spectra of the sample annealed at 300 °C. The background has been removed from all cases to prevent the contribution of the vials to the PL. This reveals the asymmetric shapes of the emission curves. Each graph includes an inset showing the vials with the diluted PSps under UV light (λ = 365 nm) to demonstrate their response to the naked eye. To investigate this asymmetrical emission, the PL signals from these graphs have been separated into two Gaussian contributions. One represents the common PS wide emission, which emits yellow–red light centered around 590 nm. The other represents the evolution of this part of the optical spectra and emits blue–green light centered around 550 nm. Table 1 shows the parameters used for the Gaussian fitting in Figure 3.

Based on the literature, the first band is known as the PS slow band (S-band) and has been proposed to be due to the quantum confinement of excitons in Si QDs [22]. The size of Si QDs is directly related to their physical dimensions, which affects the PL emission [7]. Assuming a QD spherical shape and a direct transition from conduction to valence band due to quantum confinement, the central emission at approximately 590 nm (2.1 eV) indicates an average band gap energy of 2.1 eV and corresponds to emission from 2.6 nm wide Si QDs. The blue–green emission with a central wavelength of around 550 nm corresponds to a photon energy of 2.3 eV, indicating smaller Si QDs with a mean size close to 2.3 nm. The difference in QDs size between both contributions is like the silicon native oxide [23], indicating further oxidation of the PSps. As shown in Table 1, the area under each peak clearly evolves over time. In both cases, the area under each peak increases significantly, though it appears to stabilize around day 37. The registered data and their corresponding uncertainty are inconclusive at this point, indicating that some stabilization has been achieved.

Figure 3a exhibits very low PL emission in both contributions, with almost no emission to the naked eye. Previous studies have revealed that dangling bonds are the primary cause of PL quenching in PS, since non-radiative electron recombination produced from excitons in Si QDs occurs at these surface states [24,25]. The blue–green contribution is due to the further oxidation of Si QDs, which results in their emission at shorter wavelengths. Figure 3b–d show the progression of PL enhancement over time for each contribution. As seen in the graph insets, the yellow–red contribution shows more advancement compared to the blue–green one. The progress can be attributed to the saturation of Si QDs surface states (dangling bonds), as a protective oxide forms after exposure to deionized water. The PL reaches a stable level after 5 weeks, as observed in our measurements.

**Figure 4:**
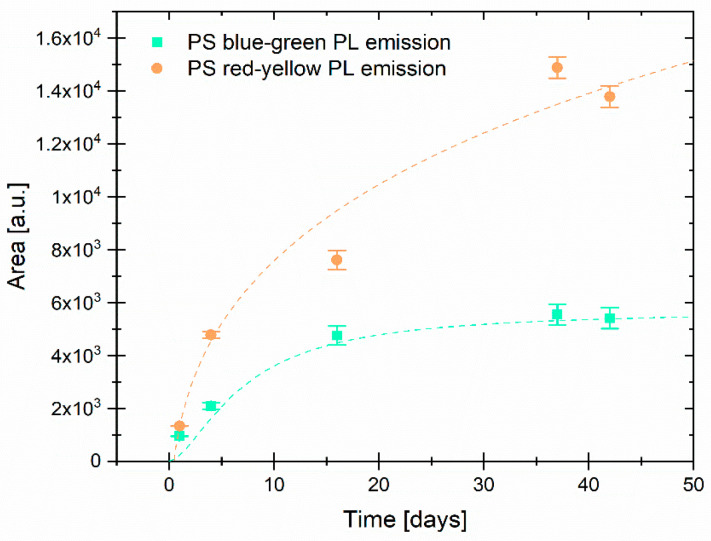
Area of the blue-green and yellow-red Gaussian contributions to the PSps PL over time, with dashed lines indicating a logistic function fit for visualization purposes.

Figure 4 displays the different PL evolutions of the blue–green and yellow–red emissions over time. The yellow–red emission increases at a faster rate than the blue–green emission during the first two weeks due to the oxidation caused by exposure to deionized water. This, in turn, leads to an increase in the number of radiative recombination events inside Si QDs [11]. Both contributions were fitted to a logistic function as a visual guide.

The sample annealed at 600 °C did not exhibit any PL during or after the PSps synthesis, which indicates that the PS layer was fully oxidized prior to nanoparticle production. The PS layers were exposed to 365 nm UV light both before and after thermal annealing to check for PL emission. The sample annealed at 300 °C exhibited decreased PL, which gradually recovered over time when exposed to deionized water. However, this recovery effect was not observed in the case of PSps produced from the PS layer annealed at 600 °C.

### 3.2. Cell Viability Assay in Presence of Porous Silicon Particles (PSps)

**Figure 5:**
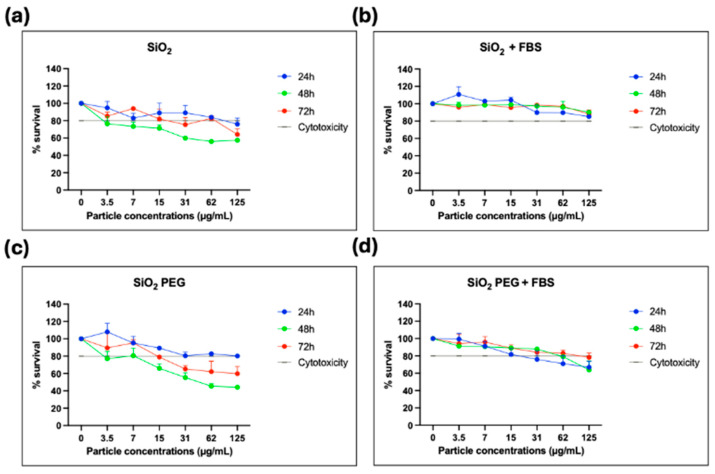
Cell viability assessed by MTT assay at 24 h (blue), 48 h (green), and 72 h (red) after exposure to PSps (3.5–125 μg/mL). (**a**) Non-PEGylated PSps in FBS-free medium. (**b**) Non-PEGylated PSps in cell medium with FBS. (**c**) PEGylated PSps in FBS-free medium. (**d**) PEGylated PSps in cell medium with FBS. The dashed line indicates the 80% viability threshold, below which cytotoxicity is considered significant. Negative controls without PSps are shown at 0 μg/mL (100% viability). Data are presented as mean ± standard error of the mean, expressed as a percentage of the negative controls, from duplicate measurements in three independent experiments.

The EaHy926 cell line is a relevant endothelial model, which has been extensively employed in nanotoxicology and vascular biology studies for initial cytotoxicity screening. Adding SiO_2_ particles to the endothelial cells in culture produced a concentration- and time-dependent decrease in the cell survival in serum-free medium (Figure 5a). This is consistent with reports on amorphous silica nanoparticles, which also induce endothelial cell death through oxidative stress, membrane damage, and pro-inflammatory signaling, especially at higher concentrations or smaller sizes [16]. Compared to dense amorphous silica, PSps offer a higher surface area and tunable porosity, which may influence both protein adsorption and dissolution dynamics, thereby modulating their biological effects.

The addition of FBS significantly reduced PSps cytotoxicity (Figure 5b; *p* < 0.0001), in agreement with previous studies showing that serum proteins rapidly adsorb to silica particles, forming a protein corona that shields reactive silanol groups, reduces membrane disruption, and alters cellular uptake [26,27]. This highlights the importance of studying nanoparticle toxicity under physiologically relevant conditions that mimic the plasma environment, i.e., a high content of proteins, hormones, and growth factors required for cell attachment and viability [27]. The significant recovery of cell viability after 72 h compared with the decrease observed at 48 h in serum-free conditions (Figure 5a; *p* < 0.05) suggests that cells may activate adaptive responses to initial nanoparticle-induced stress. Possible mechanisms include induction of antioxidant defense systems, upregulation of stress-response genes, or enhanced autophagic clearance of damaged organelles, which collectively improve survival. In parallel, silica particles may undergo aggregation or partial dissolution over time in the culture medium, thereby reducing the effective number of bioactive particles interacting with the cell surface [16].

PEGylation showed a reduction in cell viability at higher concentrations (≥15 µg/mL) with clear cytotoxic effects at longer incubation times (48 and 72 h) (Figure 5c). This effect was significant compared to non-PEGylated SiO_2_ (Figure 5a; *p* < 0.002), it showed a partial recovery after 72 h (*p* < 0.05) and was also prevented in the presence of FBS (Figure 5d; *p* < 0.005). Nonetheless, even in presence of serum, a cytotoxic effect was evident at concentrations ≥ 62 µg/mL, suggesting a PEGylation-dependent toxicity. This is surprising since PEGylation usually enhances endothelial biocompatibility through the reduction in oxidative stress and inflammation compared to unmodified PSps [28]. One plausible mechanism is that the PEG shell, particularly if not densely grafted, does not fully exclude protein adsorption but may actually alter the protein corona in a way that enhances cellular uptake, leading to a higher cell internalization. Once inside, the PEGylated particles may reside longer in endosomal and/or lysosomal compartments, promoting generation of reactive oxygen species, as well as mitochondrial or endoplasmic reticulum stress, and ultimately cell death [29].

A limitation of this study is that amorphous silica particles were not included as a control for PSps and PSpsPEG. Future comparative studies incorporating amorphous silica will be important to better contextualize the cytotoxicity of PSps and to strengthen the interpretation of their relative safety profile.

## 4. Conclusions

A two-step oxidation process was used to produce PSps with a stable and long-lasting PL emission. The first step involved annealing in air at 300 °C and 600 °C to induce the growth of a protective oxide over the PS surface. The first annealing temperature produced mild oxidation, while the second completely quenched the PL emission. In both cases, there was an oxidation gradient along the PS layer, resulting in the formation of particles with varying degrees of oxidation. The PS layers were then sonicated in an ultrasonic bath and stored in deionized water to produce the PSps. The exposure to deionized water caused further oxidation, which helped recover the PSps PL over time.

The PL emission of PSps was divided into two components: yellow–red emission attributed to Si QDs and blue–green emission representing the further oxidation of smaller Si QDs. These components evolved differently over time, with the yellow–red emission increasing at a higher rate until stabilization around 5 weeks after synthesis, while the blue–green emission stabilized a couple of weeks after the PSps synthesis.

In conclusion, a mild annealing in the air followed by further oxidation in water can help achieve long-lasting PL emissions in PSps. The initial protective oxide layer resulting from annealing at 300 °C is not enough to stabilize the entire PS layer due to inhomogeneous oxidation, but subsequent oxidation in water after sonicating the PS layer completes the PSps PL stabilization over time.

The fact that PSps synthesized in this study did not exert cytotoxic effects on human endothelial cells under conditions mimicking the physiological plasma environment supports their potential as candidates for biomedical applications such as drug delivery or cell tracking. However, the observation that PEGylation increased cytotoxicity highlights the need for further studies to clarify the underlying mechanisms, including the role of the protein corona, cellular uptake pathways, and intracellular stress responses. Additional in vitro and in vivo investigations will therefore be required to fully establish the safety profile and optimize the design of PSps for clinical use.

## Figures and Tables

**Table 1:** Parameters used for the Gaussian fitting in Figure 3.

	Yellow–Green Emission			Yellow-Red–Emission		
Day	Peak Position [nm]	Area [a.u.]	FWHM [nm]	Peak Position [nm]	Area [a.u.]	FWHM [nm]
1	542.8 ± 0.2	957 ± 8	47.3 ± 0.4	597 ± 1	1330 ± 10	78.0 ± 0.7
4	551.8 ± 0.3	2100 ± 100	46.8 ± 0.8	593 ± 1	4800 ± 100	79 ± 1
16	554.7 ± 0.4	4800 ± 400	50.5 ± 0.9	594 ± 2	7600 ± 400	75 ± 2
37	554.3 ± 0.3	5500 ± 400	49.2 ± 1.0	594 ± 1	14,900 ± 400	80 ± 1
42	555.9 ± 0.3	5400 ± 400	48.3 ± 1.0	595 ±1	13,800 ± 400	79 ± 1

## Data Availability

The original contributions presented in this study are included in the article. Further inquiries can be directed to the corresponding author(s).

## Abbreviations

The following abbreviations are used in this manuscript:

EDX	Energy-Dispersive X-Ray Spectroscopy
FBS	Fetal Bovine Serum
PEG	Polyethylene Glycol
PL	Photoluminescence
PS	Porous Silicon
PSps	Porous Silicon Particles
QDs	Quantum Dots
SEM	Scanning Electron Microscopy
UV	Ultraviolet

## References

[1] Cullis A., Canham L. (1991). Visible light emission due to quantum size effects in highly porous crystalline silicon. Nature.

[2] Anglin E.J., Cheng L., Freeman W.R., Sailor M.J. (2008). Porous silicon in drug delivery devices and materials. Adv. Drug Deliv. Rev..

[3] Park J.-H., Gu L., von Maltzahn G., Ruoslahti E., Bhatia S.N., Sailor M.J. (2009). Biodegradable luminescent porous silicon nanoparticles for in vivo applications. Nat. Mater..

[4] Lockwood D.J., Wang A.G. (1995). Quantum confinement induced photoluminescence in porous silicon. Solid State Commun..

[5] Jung Y., Kang S., An J., Jung J., Kim D. (2020). Porous silicon-based fluorescent nanoprobe for the detection of anthrax biomarker and its practical sensing applications. Dyes Pigments.

[6] Delerue C., Allan G., Lannoo M. (1993). Theoretical aspects of the luminescence of porous silicon. Phys. Rev. B.

[7] Pérez M., Ramos E., de la Mora M.B., Santana G., Dutt A. (2021). Absorption and emission of porous silicon based on quantum dots models by TD-DFT: Experimental and theoretical approach. Mater. Lett..

[8] Gole J.L., Dudel F.P., Grantier D., Dixon D.A. (1997). Origin of Porous Silicon Photoluminescence: Evidence for a Surface Bound Oxyhydride-Like Emitter. Phys. Rev. B.

[9] Gole J.L., Dixon D.A. (1998). Potential Role of Silanone and Silylenes in the photoluminescence-excitation, visible-photoluminescence-emission, and infrared spectra of porous silicon. Phys. Rev. B.

[10] Sailor M.J., Canham L. (2014). Chemical Reactivity and Surface Chemistry of Porous Silicon. Handbook of Porous Silicon.

[11] Nakamura T., Ogawa T., Hosoya N., Adachi S. (2010). Effects of thermal oxidation on the photoluminescence properties of porous silicon. J. Lumin..

[12] Björkqvist M., Salonen J., Laine E., Niinistö L. (2023). Comparison of stabilizing treatments on porous silicon for sensor applications. Phys. Status Solidi (A) Appl. Mater. Sci..

[13] Boukherroub R., Wayner D.D.M., Sproule G.I., Lockwood D.J., Canham L.T. (2001). Stability enhancement of partially-oxidized porous silicon nanostructures modified with ethyl undecylenate. Nano Lett..

[14] Petrova-Koch V., Muschik T., Kux A., Meyer B.K., Koch F., Lehmann V. (1992). Rapid-thermal-oxidized porous Si-The superior photoluminescent Si. Appl. Phys. Lett..

[15] Duan J., Yu Y., Li Y., Yu Y., Li Y., Zhou X., Huang P., Sun Z. (2013). Toxic effect of silica nanoparticles on endothelial cells through DNA damage response via Chk1-dependent G2/M checkpoint. PLoS ONE.

[16] Napierska D., Thomassen L.C.J., Rabolli V., Lison D., Gonzalez L., Kirsch-Volders M., Martens J.A., Hoet P.H. (2009). Size-dependent cytotoxicity of monodisperse silica nanoparticles in human endothelial cells. Small.

[17] Bauer A.T., Strozyk E.A., Gorzelanny C., Westerhausen C., Desch A., Schneider M.F., Schneider S.W. (2011). Cytotoxicity of silica nanoparticles through exocytosis of von Willebrand factor and necrotic cell death in primary human endothelial cells. Biomaterials.

[18] Corbalan J.J., Medina C., Jacoby A., Malinski T., Radomski M.W. (2011). Amorphous silica nanoparticles trigger nitric oxide/peroxynitrite imbalance in human endothelial cells: Inflammatory and cytotoxic effects. Int. J. Nanomed..

[19] Jacobsohn L.G., Cooke D.W., Bennett B.L., Muenchausen R.E., Nastasi M. (2005). Effects of thermal annealing and ageing on porous silicon photoluminescence. Philos. Mag..

[20] Shin H.J., Lee M.K., Hwang C.C., Kim K., Kang T.H., Kim B., Kim G.B., Hong C.K., Lee K.-W.W., Kim Y.Y. (2003). Photoluminescence degradation in porous silicon upon annealing at high temperature in vacuum. J. Korean Phys. Soc..

[21] Mosmann T. (1983). Rapid colorimetric assay for cellular growth and survival: Application to proliferation and cytotoxicity assays. J. Immunol. Methods.

[22] Rodríguez J.A., Vásquez-Agustín M.A., Morales-Sánchez A., Aceves-Mijares M. (2014). Emission Mechanisms of Si Nanocrystals and Defects in SiO_2_ Materials. J. Nanomater..

[23] Morita M., Ohmi T., Hasegawa E., Kawakami M., Ohwada M.J. (1990). Growth of native oxide on a silicon surface. Appl. Phys..

[24] Shimasaki M., Show Y., Iwase M., Izumi T., Ichinohe T., Nozaki S., Morisaki H. (1996). Correlation between light emission and dangling bonds in porous silicon. Appl. Surf. Sci..

[25] Ookubo N., Ono H., Ochiai Y., Mochizuki Y., Matsui S. (1992). Effects of thermal annealing on porous silicon photoluminescence dynamics. Appl. Phys. Lett..

[26] Docter D., Bantz C., Westmeier D., Galla H.J., Wang Q., Kirkpatrick J.C., Nielsen P., Maskos M., Stauber R.H. (2014). The protein corona protects against size- and dose-dependent toxicity of amorphous silica nanoparticles. Beilstein J. Nanotechnol..

[27] Mahmoud N., Abu-Dahab R., Abdallah M., Al-Dabash S., Abuarqoub D., Albasha A., Khalil E.A. (2020). Interaction of gold nanorods with cell culture media: Colloidal stability, cytotoxicity and cellular death modality. J. Drug Deliv. Sci. Technol..

[28] Liang S., Chen Y., Zhang S., Cao Y., Duan J., Wang Y., Sun Z. (2020). RhB-encapsulating silica nanoparticles modified with PEG impact the vascular endothelial function in endothelial cells and zebrafish model. Sci. Total Environ..

[29] Baghirov H., Karaman D., Viitala T., Duchanoy A., Lou Y.-R., Mamaeva V., Pryazhnikov E., Khiroug L., de Lange Davies C., Sahlgren C. (2016). Feasibility study of the permeability and uptake of mesoporous silica nanoparticles across the blood-brain barrier. PLoS ONE.

